# Music in healthcare: Investigating music preferences for pain management across twenty countries

**DOI:** 10.1016/j.ssmph.2025.101758

**Published:** 2025-01-22

**Authors:** Antonia S. Becker, Emy S. van der Valk Bouman, Julian Schaap, Markus Klimek, Joost Oude Groeniger

**Affiliations:** a Department of Neuroscience, Erasmus Medical Center, Rotterdam, the Netherlands; b Department of Arts and Culture Studies, Erasmus University, Rotterdam, the Netherlands; c Department of Anesthesiology, Erasmus Medical Center Rotterdam, Rotterdam, the Netherlands; d Department of Public Health, Erasmus Medical Center, Rotterdam, the Netherlands

## Abstract

Music is increasingly investigated in healthcare to manage pain, anxiety and stress. Previous studies have demonstrated that listening to (preferred) music is effective for pain relief, but rarely explore whether people are open to instrumentalizing music in healthcare. Therefore, this study investigates 1) to what extent people across twenty countries are willing to listen to music when experiencing pain in healthcare, and 2) which music genres they choose, in light of their national context, background characteristics, and overall music preferences. In addition, we investigate the universality of the so-called ‘Mozart effect’, which describes the belief that classical music is superior in healthcare, despite research suggesting that favorite music, irrespective of genre, is most effective. To answer these questions, we use data from the International Federation of the Phonographic Industry's international survey (2023), which includes twenty countries and 33,629 participants. In total, a large majority (86.5%) wants to listen to music when experiencing pain in healthcare. Although participants predominantly select music in line with their favorite music genres (73.1%), a smaller trend toward selecting classical music – in line with the ‘Mozart effect’ – is observed. Among those who prefer to listen to a music genre that they did not list as their favorite, classical music is predominantly chosen (43.3%). Furthermore, there are notable differences between national populations and across social groups in terms of preference for music when experiencing pain. These insights can be crucial for implementing music in healthcare worldwide, emphasizing the need for a culturally sensitive and personalized approach.

## Highlights

• A large majority (86.5%) of people prefer listening to music when experiencing pain.
• Most people choose to listen to their favorite music for pain relief (73.1%).
• The willingness to listen to music for pain relief varies notably across countries.
• The belief that classical music better relieves pain (‘Mozart effect’) is present.

## 1 Introduction

With its deep evolutionary origins, music is part of every known human society (Ian & Morley, 2010). Its broad definition varies across cultures and scientific disciplines. In medicine, music has long been recognized for its potential therapeutic effects, particularly in the context of pain management (Hole et al., 2015). In line with its positive influence on pain, anxiety and stress, previous research has indicated a lower requirement for opioids when patients listen to music during their hospital stay (Fu et al., 2020). Moreover, a recent report by the World Health Organization emphasized the importance of arts in healthcare (Fancourt & Finn, 2019). This underscores the need to facilitate music and other non-pharmacological measures as therapeutic tools in healthcare, and to intensify research efforts to understand their properties and effects.

Several studies have investigated the attitudes of healthcare professionals toward the instrumentalization of music in healthcare, highlighting the positive effects of music for their patients (Khan et al., 2016; Rodríguez-Rodríguez et al., 2024). However, no research has been conducted on the perception of the general population, evaluating whether patients want to listen to music at all and, if so, which kind of music they would like to listen to. Despite expanding research on the therapeutic effects of music in recent years, there remains a significant gap in our understanding of how people from diverse socio-cultural backgrounds perceive its role in healthcare (Becker et al., 2024). While individual studies have examined cultural differences in music preferences for pain relief in different countries (Lim & Locsin, 2006; Tola et al., 2022), there is no overarching study that looks at music as medicine from a global perspective. Furthermore, extant studies have not investigated whether music preferences when experiencing pain also vary within national populations. Since gender, age, income, and ethno-racial differences are known to influence taste preferences (Bourdieu, 1984; Lizardo & Skiles, 2016; Van Eijck, 2001), it is important to include these differences in such an analysis.

This gap in knowledge is further complicated by the diverse range of music selections used in existing studies on music as medicine (Hole et al., 2015). Currently, there is a noticeable lack of guidelines on how to facilitate the use of music in healthcare, particularly in determining which type of music should be offered to which patients. Medical research on music in healthcare partially focuses on identifying relatively ‘universal’ kinds of music that are effective for most people. This is understandable, since identifying such music would have great benefits in healthcare, simplifying the process of music selection for various patients. Aside from research into physiological responses toward certain kinds of frequencies through ambient sonic soundscapes or ‘binaural’ electronic music (Garcia-Argibay et al., 2019; Gkolias et al., 2020), ample research has focused on Western classical music (Hole et al., 2015). The potential universal effectiveness of Western classical music is encapsulated in the so-called ‘Mozart-effect’, a widely mediatized idea that Western classical music – Mozart's music in particular – is universally beneficial to people, from newborn babies to people in elderly care homes (Pietschnig et al., 2010).

The term ‘Mozart effect’ emerged from a 1993 study by Rauscher et al. in which participants listened to Mozart's Sonata for Two Pianos in D major, K. 448, and then completed spatial reasoning tasks (Rauscher et al., 1993). The results revealed a temporary enhancement in spatial reasoning skills. Following this, Mozart's music has been extensively investigated in healthcare-related contexts, including research on dementia, cardiovascular disorders and epilepsy (Pauwels et al., 2014). Even though the superior effect of classical and/or Mozart's music alone is not proven – especially not in comparison to other types of music– research continues to focus on Mozart's sonata K. 448 and related classical music compositions (Oberleiter & Pietschnig, 2023). However, recent studies investigating the effectiveness of music in pain management have demonstrated that people's (socially differentiated) music preferences are stronger indicators of music's effectiveness than specific kinds of music (Van der Valk Bouman et al., 2024). Moreover, a recent randomized controlled trial revealed that self-chosen music had greater effects on anxiety, blood pressure and overall satisfaction as compared to Mozart's K. 448 within the perioperative setting (Barlas et al., 2023). In other words, research indicates that music is more effective when it is in line with the listener's taste, irrespective of genre. Despite this, owing to the widespread belief in the universal effectiveness of Western classical ‘highbrow’ music, patients (and medical practitioners) may favor this music over their preferred music in healthcare (Van der Valk Bouman et al., 2024). To date, our understanding of (the persistence of) this perspective remains limited, especially when looking at cultural variations across different countries.

Previous research has demonstrated that people's background plays a crucial role in shaping music taste formation. Therefore, being sensitive to such differences is important. First, socio-economic status has historically been a strong indicator of music taste patterns (Bourdieu, 1984; Lizardo & Skiles, 2016; Van Eijck, 2001). While people have become increasingly omnivorous in their preferences for different genres across the ‘highbrow-lowbrow’ spectrum, this remains more pronounced among middle-class groups than among lower- and upper-class groups (Nault et al., 2021; Reeves, 2019). Among more educated people, preferences for classical music and dislikes for popular music tend to be more prevalent than among people with less-educated backgrounds, although this varies across national contexts (Atkinson, 2011; Reeves, 2019). This is important, because socioeconomic status is also a strong determinant of health and healthcare outcomes (Mackenbach, 2019). Indeed, those with a higher socioeconomic status generally live longer and have better health across the life course (Marmot et al., 2008). Various resources contribute to the more advantageous health status of higher socioeconomic groups, including cultural resources (i.e., cultural capital) that enable adopting healthier lifestyles and navigating health care systems more effectively (Dubbin et al., 2013; Mudd et al., 2023; Williams, 1995). Most notably, it has been shown that the tastes and dispositions acquired in privileged positions linked to a preference for ‘highbrow’ music taste are also linked to better health outcomes (Oude Groeniger et al., 2020). Second, gender can influence musical preferences, with women on average favoring genres such as classical and chart pop, whereas men tend to lean more toward ‘heavier’ genres such as rock, metal or electronic music (Bennett et al., 2009; Colley, 2008). Third, age-related differences impact musical tastes, with older adults appreciating classical music and younger generations gravitating toward contemporary genres (Bonneville-Roussy et al., 2013; Glevarec et al., 2020). Finally, ample research has demonstrated how ethno-racial differences, within and between national contexts, can affect preferences for music tastes (Lizardo & Skiles, 2016; Schaap et al., 2022).

In summary, while musical tastes clearly vary across different backgrounds, the factors influencing the selection of music for pain relief in the global population remain unclear. The International Federation of the Phonographic Industry (IFPI) has empirically captured music trends across countries for an extensive period and is considered authoritative within the global music industry (Engaging with Music 2023). By collaborating with the IFPI in their annually conducted Music Engagement Report, we had the possibility to work with unique data to answer our questions about music in healthcare across various countries across the globe. The aims of this study were defined as 1) to what extent people across twenty countries are willing to listen to music when experiencing pain in healthcare, and 2) to explore which music genres they choose, considering their national context, personal background characteristics and overall music preference. By extension, we intend to investigate the presence of the ‘Mozart effect’, i.e., whether people rather select classical music instead of their preferred music genre when experiencing pain. By answering these questions, we aim to enhance our understanding of how the global population perceives music as medicine and provide new insights for its implementation in healthcare.

## 2 Methods

### 2.1 Participants

The IFPI's Music Consumer Study 2023 was a multi-country research study focused on music habits (Engaging with Music 2023). The study comprised an international cross-sectional survey across twenty-six countries in 2023 and involved 43,500 respondents. Certain questions were restricted to specific countries. The question ‘music as medicine’, which involved music listening in healthcare when experiencing pain, was asked to all respondents in Australia, Canada, China, France, Germany, India, Italy, Japan, the Netherlands, New Zealand, Nigeria, Poland, Saudi Arabia, South Africa, South Korea, Spain, Sweden, the United Arabic Emirates (UAE), the United Kingdom (UK), and the United States of America (USA).

Panels in each country were selected to be nationally representative of the online population according to the most recent census data. An overview of panel sizes, panel age ranges and panel providers per country can be found in Supplementary Table 1. Field work and data collection took place between August and October 2023.

### 2.2 Survey and procedure

The complete survey was designed by the IFPI's Insight and Analysis Department, with an intended length of 20 min. Informed consent was obtained before the survey was completed. The survey was administered online. The IFPI commissioned a research agency (AudienceNet) to script the survey using Qualtrics, locate suitable panel providers for each country, ensure that sufficient respondents completed the full questionnaire in each market, and process the resulting data. The data were anonymized by the IFPI before being transferred.

Our research group had the possibility of incorporating one question in the IFPI's 2023 survey. This question was based on expert opinion and previous literature, combined with knowledge and survey construction possibilities as decided by the IFPI team. The question was discussed and adjusted until consensus was reached. The final question was “If you were in a hospital or other healthcare setting and experiencing pain, what type of music would you most like to listen to? Please select one choice only”. There were ten multiple-choice options that participants could select, partly based on participants' previous choices regarding overall music genre preference, and an open answer option. An overview of the question with its answer options can be found in Supplementary Table 2. Answer options of the demographic characteristics (age, gender, ethnicity, income) are shown in Supplementary Table 3.

Answers written down as free text in the open-answer option for both the music when experiencing pain and the favorite music genres questions resulted in 1206 specific genres. These were subsequently categorized into widely established genre clusters (Franken et al., 2017; Musicmap. Version 1.2.0. 2022; Rentfrow et al., 2011). Two researchers (AB, EVB) read through all open answers (1206), and categorized them independently, and discussed them with a third researcher (JS) in case of doubt. The categories were then slightly expanded on the basis of previous literature. Finally, agreement on eighteen genre groups, including one group ‘No music at all’ and one group ‘Other’ (with genres that could not be placed in one genre group such as radio, white noise and a mix of music), was reached. Supplementary Table 4 provides an overview of the genre groups with the subgenre categorization that was used in this study.

The genre choice when experiencing pain was compared with the overall genre preference, as indicated at an earlier question in the survey. Participants could choose at least one and up to three favorite genres (first, second, and third preferences). When these genres overlapped with the music genre chosen when experiencing pain, it was categorized as ‘favorite music’. All other choices were categorized as ‘non-favorite music’, i.e., cases where the genre chosen when experiencing pain was different from any of the preferred genres. The category ‘No music at all’ was kept separate from this categorization.

### 2.3 Data analysis

Data were analyzed and visualized using Stata (version 18), R-Studio (version 2023.12.0) and R (version 4.3.2) with the following packages: dplyr, tidyr, ggplot2, ggbeeswarm, car, broom and GLMMadaptive. For continuous variables, the mean and standard deviation (SD) were used, whereas categorical variables were summarized using percentages and numbers. These statistics were weighted based on the number of participants from each country. In this way, each country was given equal weight in the overall descriptive analysis, ensuring fair representation regardless of the sample size from each country.

First, we used logistic multilevel regression models to examine the relationships of gender and age with preferences for music when experiencing pain across the twenty countries. We assessed i) the choice of no music vs. music, and ii) the choice of non-favorite genre vs. favorite genre (excluding the category ‘No music at all’). In this model, countries were included as a random intercept. Gender was measured as male, female or other. Age groups (16–24; 25–34; 35–44; 45–54; 55–64; 65+) were created as categorical variables because of their nonlinear relationship with the choice of music. The 65+ age group was present only in France and the Netherlands (Supplementary Table 1). Second, we used logistic regression models to examine the relationships of income and race-ethnicity with preferences for music when experiencing pain in a subgroup analysis focusing on the UK and USA, as these variables were only available for these two countries. The analysis was conducted separately for both countries because of the different categorizations of income and race-ethnicity, and were adjusted for age and gender. Third, to investigate the ‘Mozart effect’, we analyzed the probability of choosing classical music vs. other music genres in the subgroup selecting non-favorite music genres. In line with the analysis mentioned above, a logistic multilevel model was used with gender and age as independent variables, based on which predicted probabilities were calculated and plotted.

## 3 Results

In total, 33,629 participants answered the question ‘music as medicine’. The survey was conducted across 20 countries, with the number of participants ranging from 743 to 4000 per country (see Table 1). The overall mean age was 38.4 years (SD = 13.8) and ranged from 16 to 95 years old. Mean age ranged from 30.0 (SD = 6.6) in Nigeria to 48.8 years (SD = 17.1) in the Netherlands. The overall distribution of female and male participants was generally balanced (49.2% female and 50.5% male).

**Table 1:** Baseline characteristics of the participating countries.

Country	N	Age mean (SD)	Male^a^ % (n)	Female^a^ % (n)	Other^a^ % (n)
Australia	1535	38.7 (13.7)	46.7 (715)	53.2 (815)	0.1 (1)
Canada	1542	40.3 (13.8)	49.6 (762)	50.2 (771)	0.1 (2)
China	3020	30.1 (7.2)	51.6 (1557)	48.2 (1456)	0.2 (5)
France	2042	47.7 (18.1)	48.2 (983)	51.8 (1057)	0.0 (0)
Germany	1502	42.5 (12.9)	47.4 (712)	52.5 (789)	0.1 (1)
India	3004	27.7 (7.17)	63.4 (1901)	36.6 (1099)	0.0 (0)
Italy	1514	42.0 (13.5)	47.2 (712)	52.6 (794)	0.3 (4)
Japan	1593	41.5 (13.8)	53.5 (849)	46.4 (736)	0.1 (1)
The Netherlands	1419	48.8 (17.1)	47.2 (669)	52.8 (748)	0.0 (0)
New Zealand	1111	37.7 (13.5)	42.8 (473)	57.1 (631)	0.2 (2)
Nigeria	743	30.0 (6.6)	61.3 (455)	38.5 (286)	0.1 (1)
Poland	1110	39.8 (12.7)	48.9 (542)	50.9 (564)	0.2 (2)
Saudi Arabia	1128	31.5 (6.31)	46.6 (523)	53.3 (598)	0.2 (2)
South Africa	1130	34.8 (12.2)	48.6 (547)	51.3 (578)	0.1 (1)
South Korea	1546	43.2 (11.6)	51.0 (788)	48.9 (756)	0.1 (2)
Spain	1533	41.0 (12.7)	48.9 (749)	50.9 (779)	0.2 (3)
Sweden	1525	42.4 (12.6)	47.3 (720)	52.6 (801)	0.1 (2)
UAE	1108	32.5 (7.0)	56.0 (618)	43.9 (484)	0.1 (1)
UK	1524	40.0 (14.0)	48.7 (739)	50.8 (772)	0.5 (8)
USA	4000	40.5 (13.8)	49.2 (1966)	50.8 (2027)	0.0 (1)
Total	33629	38.4 (13.8)	50.2 (16980)	49.7 (16541)	0.1 (40)

Abbreviations: SD = standard deviation, UAE = United Arabic Emirates, UK = United Kingdom, USA = United States of America.

a Gender frequencies were calculated excluding the option ‘Prefer not to say’ (overall n = 68; 0.2%).

### 3.1 Preference to listen to music when experiencing pain

**Fig. 1:**
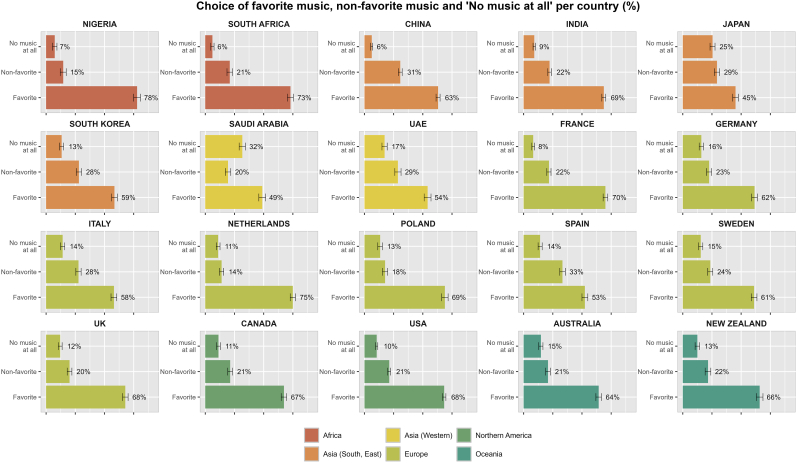
Choice of favorite music, non-favorite music and no music at all per country (%) Abbreviations: UAE = United Arab Emirates, UK = United Kingdom, USA = United States of America.

The choice of music genre when experiencing pain was compared to the participants' favorite music genres. Music genres not represented in the participants' top three favorite music genres were categorized as ‘non-favorite’. Overall, 13.5% of all participants chose ‘No music at all’ when experiencing pain in healthcare (Fig. 1). The lowest percentages were observed in South Africa (6.1%) and China (6.3%), whereas the highest percentages were observed in Saudi Arabia (31.7%) and Japan (25.5%). Across all 20 countries, participants predominantly chose their favorite music when experiencing pain, with percentages ranging from 45.2% (Japan) to 77.9% (Nigeria), with an average of 63.5%. In contrast, non-favorite music was selected on average by 23.0% of all participants, ranging from 14.0% (the Netherlands) to 33.3% (Spain).

The impact of age and gender on the choice of music (no music vs. music; non-favorite vs. favorite music genres) was examined through a logistic multilevel analysis (Table 2). Female participants were more likely to choose either no music (OR 0.81, 95% CI 0.76–0.81) or a genre other than their favorite music genre (OR 0.93, 95% CI 0.88–0.93) when experiencing pain compared to male participants. Compared to the youngest group (16–24 years), participants between 25 and 34 years of age had a greater probability (OR 1.16, 95% CI 1.05–1.29) of choosing to listen to music, whereas the older age groups (≥45 years) had a lower probability (45–54 years: OR 0.88, 95% CI 0.79–0.99; 55–64 years: OR 0.81, 95% CI 0.72–0.91; 65+ years: OR 0.73, 95% CI 0.56–0.95). In particular, the older (≥55 years) groups had a significantly greater probability of choosing music genres when experiencing pain that align with their favorite music genre(s) in comparison to the youngest age group (55–64 years: OR 1.24, 95% CI 1.21–1.37; 65+ years: OR 1.40, 95% CI 1.12–1.75).

**Table 2:** Relationships between gender and age and music choice across twenty countries.

	No music (0) vs. Music (1)		Non-favorite (0) vs. Favorite music genres (1)^a^	
	OR	95% CI	OR	95% CI
Gender - female	0.81	0.76–0.87	0.93	0.88–0.98
Age (years)				
16–24 (n = 6474)	(ref.)	(ref.)	(ref.)	(ref.)
25–34 (n = 8923)	1.16	1.05–1.29	1.02	0.94–1.10
35–44 (n = 7688)	0.94	0.85–1.05	0.97	0.90–1.05
45–54 (n = 5063)	0.88	0.79–0.99	1.07	0.97–1.18
55–64 (n = 4648)	0.81	0.72–0.91	1.24	1.12–1.37
65+ (n = 833)^b^	0.73	0.56–0.95	1.40	1.12–1.75

Abbreviations: CI = confidence interval; ref. = reference category; OR = odds ratio.

a Excluding ‘No music at all’.

b Group only in France and the Netherlands.

To investigate the relationships of income and race-ethnicity with the choice of music (no music vs. music; non-favorite vs. favorite music genres), we conducted a subanalysis for the UK and USA (Table 3). A comprehensive overview of the baseline characteristics for these two countries, including income and race-ethnicity, is provided in Supplementary Table 5. In the UK, participants of Asian descent had a greater probability of selecting no music than music (OR 0.56, 95% CI 0.33–0.97) compared to White participants. Additionally, participants with an income below average (less than £20,000) were marginally less inclined to select music when experiencing pain (OR 0.65, 95% CI 0.42–1.00) compared to participants with an income of £20,000 - £39,999. In the USA, Black participants were more likely to select their favorite music genres (OR 1.38, 95% CI 1.07–1.79) compared to White participants. Moreover, participants with an income above average (more than $50,000) had a greater probability of selecting non-favorite music genres ($50,000–74,999: OR 0.70, 95% CI 0.55–0.89; $75,000–99,999: OR 0.72, 95% CI 0.55–0.94; more than $100,000: OR 0.62, 95% CI 0.49–0.78) compared to participants with an income of $25,000–49,999. Additionally, within the USA subset, participants from the highest income category (more than $100,000) were more likely to select music when experiencing pain (OR 1.51, 95% CI 1.10–2.07) compared to participants with an income of $25,000–49,999.

**Table 3:** Relationships between race-ethnicity and income and music choice in the UK and USA.

	No music (0) vs. Music (1)		Non-favorite (0) vs. Favorite music genres (1)^a^	
	OR	95% CI	OR	95% CI
**UK (n=1524)**				

Race-ethnicity				
White (n = 1267)	(ref.)	(ref.)	(ref.)	(ref.)
Black (n = 57)	1.78	0.54–5.87	0.81	0.42–1.57
Asian (n = 143)	0.56	0.33–0.97	0.80	0.51–1.26
Mixed (n = 26)	0.83	0.19–3.76	0.87	0.27–2.78
Other (n = 12)	0.46	0.09–2.27	0.53	0.12–2.27
Income				
Less than £20,000 (n = 265)	0.65	0.42–1.00	1.05	0.70–1.57
£20,000 - £39,999 (n = 512)	(ref.)	(ref.)	(ref.)	(ref.)
£40,000 - £59,999 (n = 304)	0.93	0.59–1.46	0.77	0.54–1.10
More than £60,000 (n = 287)	1.59	0.92–2.73	1.01	0.70–1.47

**USA (n=4000)**				

Race-ethnicity				
White (n = 2610)	(ref.)	(ref.)	(ref.)	(ref.)
Black (n = 550)	0.95	0.69–1.29	1.38	1.07–1.79
Asian (n = 244)	0.81	0.53–1.23	0.88	0.64–1.21
Hispanic/Latino (n = 201)	1.06	0.63–1.78	1.23	0.85–1.79
Mixed (n = 328)	1.14	0.75–1.75	1.04	0.78–1.40
Other (n = 47)	1.11	0.39–3.15	0.70	0.35–1.40
Income				
Less than $24,999 (n = 754)	1.06	0.80–1.44	0.97	0.75–1.25
$25,000–49,999 (n = 967)	(ref.)	(ref.)	(ref.)	(ref.)
$50,000–74,999 (n = 805)	1.23	0.91–1.67	0.70	0.55–0.89
$75,000–99,999 (n = 516)	1.30	0.91–1.85	0.72	0.55–0.94
More than $100,000 (n = 876)	1.51	1.10–2.07	0.62	0.49–0.78

All the models were also adjusted for age and gender. For both race-ethnicity and income the largest category was used as the reference category.

Abbreviations: CI = confidence interval; ref. = reference category; OR = odds ratio; UK = United Kingdom; USA = United States of America.

a Excluding ‘No music at all’.

### 3.2 Music genre selection

**Fig. 2:**
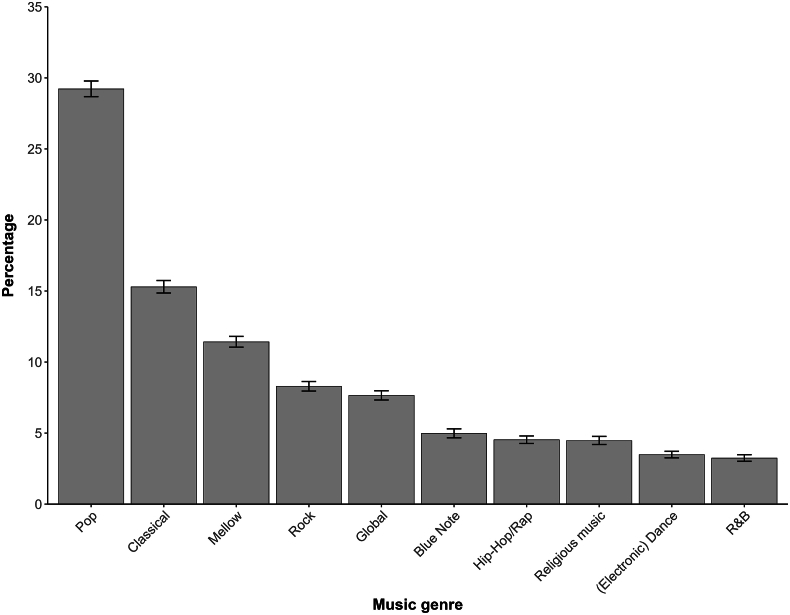
Music genre choice when experiencing pain across 20 countries The top 10 out of 17 genres are shown, excluding ‘No music at all’. Statistics were adjusted for the number of participants from each country. The weighted average and 95% coinfindence intervals were calculated based on the percentages from the 20 countries.

Fig. 2 shows the overall music genre choices across the 20 countries, corrected for the number of participants per country. Only the 10 most frequently chosen genres are depicted. Pop music was predominantly chosen (29.2%), after which classical (15.2%), mellow (11.4%), rock (8.3%) and global (7.7%) music followed. Supplementary Fig. 1 shows the five most frequent choices when experiencing pain per county. Pop music was chosen predominantly in regions such as Europe, North America, and Oceania, whereas Western Asian countries such as Saudi Arabia and the UAE most frequently chose classical music. Blue note music, such as blues, gospel and jazz, was the most common genre chosen when experiencing pain in African countries (Nigeria, South Africa).

**Fig. 3:**
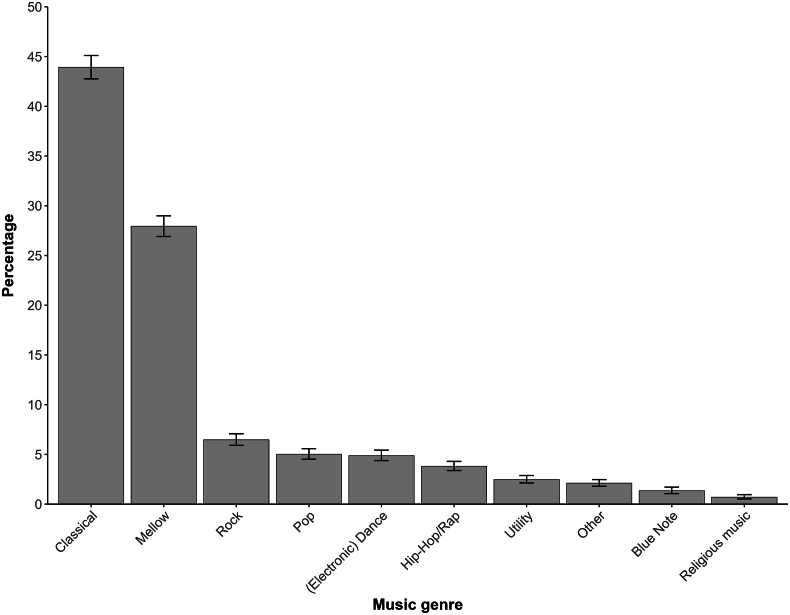
Non-favorite music genre choice when experiencing pain across 20 countries The top 10 out of 17 genres are shown. Statistics were adjusted for the number of participants from each country. The average and 95% coinfindence intervals were calculated based on the percentages from the 20 countries.

The music genre choice of participants not selecting (one of) their favorite music genre(s) is visualized in Fig. 3, with correction for the number of participants per country. Overall, classical music (43.9%) was the most common music genre. In addition, mellow (27.9%) and utility (2.5% in comparison to 0.7% in the overall choice) music, such as meditation, relaxing and healing music, were more strongly represented in this choice. The highest percentage of classical music was chosen in South Korea (72.9%), followed by Japan (72.2%) and Saudi Arabia (57.3%). Supplementary Fig. 2 provides a detailed overview of the five genres that were selected most under ‘non-favorite’ music per country. These findings suggest that a substantial number of people would select music (classical and mellow specifically) that is not in line with their personal preference when experiencing pain.

**Fig. 4:**
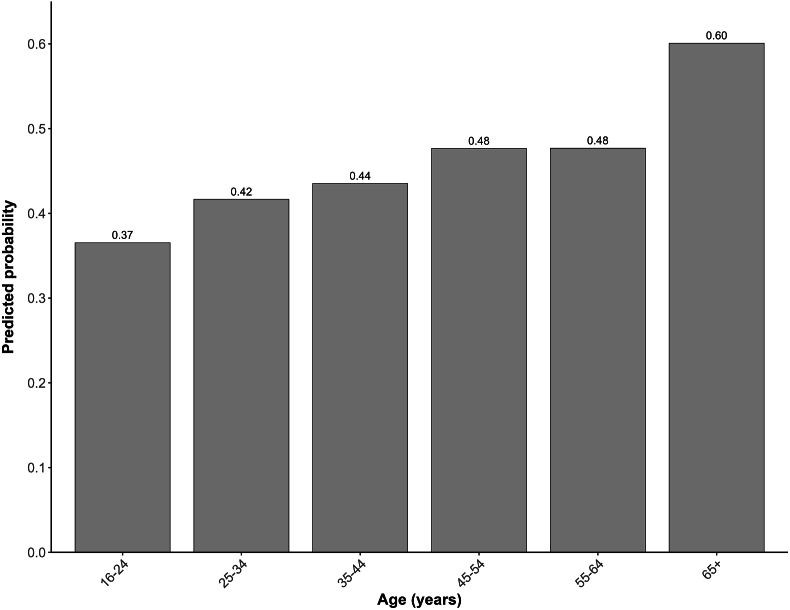
Predicted probability per age group to choose classical music under 'Non-favorite' Predicted probability of choosing classical music under participants selecting ‘Non-favorite’ music genres per age group. The predicted probabilities were calculated based on a logistic multilevel analysis with gender and age as independent variables (reference category: 16–24 years). The 65+ age group was only based on data from France and the Netherlands.

Subsequently, the impact of age and gender on the choice of classical music in comparison with other music genres was examined. Logistic multilevel regression was used, and the predicted probabilities were calculated within the group of participants selecting ‘Non-favorite’ music. Gender had no discernible influence on this choice (OR 1.01, 95% CI 0.92–1.11), whereas increased age was associated with an increased probability of choosing classical music when experiencing pain (Fig. 4). Most notably, the oldest age group (65+ years) had a 23.6% greater probability of choosing classical music compared to the youngest age group (16–24 years: reference category; 25–34 years: OR 1.24, 95% CI 1.08–1.43; 35–44 years: OR 1.34, 95% CI 1.16–1.55; 45–54 years: OR 1.58, 95% CI 1.33–1.87; 55–64 years: OR 1.58, CI 1.32–1.90; 65+ years: OR 2.61, 95% CI 1.72–3.96).

## 4 Discussion

**Fig. 5:**
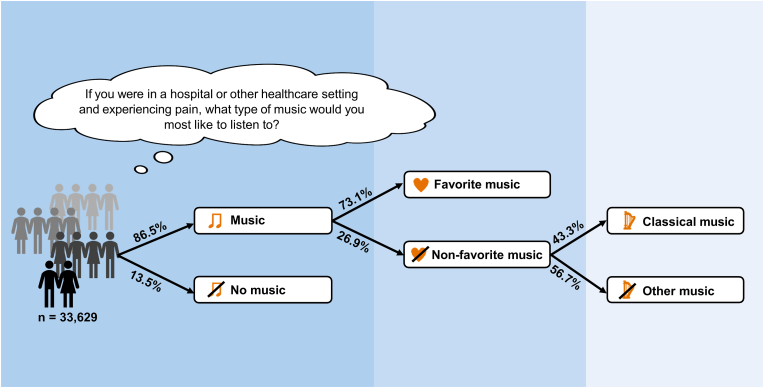
Graphical summary of key findings In total, 33,629 participants answered to the question ‘music as medicine’. Participants could select one music genre, including the option ‘No music at all’. The figure shows the average percentages looking at music vs. no music, favorite vs. non-favorite music and classical music vs. other music genres.

The aims of this study were to investigate whether participants from twenty different countries want to listen to music when experiencing pain, and to explore which music genres they choose, considering national context, background characteristics and overall music preference. Our findings indicate that across all countries, a large majority (86.5%) wants to listen to music when experiencing pain in healthcare. When looking at the music choice not in line with the personal music preferences, almost half of the participants chose classical music (43.3%). This can be taken as evidence supporting the existence of the ‘Mozart effect’ across different countries, although the majority of participants selected favorite music genres (73.1%), as recommended based on previous research. There were notable differences between national populations and across social groups (gender, age, race-ethnicity, income) in terms of preference for music when experiencing pain. The key findings of this study are summarized in Fig. 5.

### 4.1 Preference to listen to music when experiencing pain

Currently, research recommends listening to music in healthcare. However, the willingness of the general population to listen to music for pain relief has not been evaluated until now. The findings of this study reveal that a large majority of the global population wants to listen to music when experiencing pain. This high percentage underscores the widespread acceptance and intuitive appeal of music as a coping mechanism and treatment option for pain management, and supports the integration of music in healthcare. Our findings also indicate that a notable minority of people (13.5%) would not want to listen to music when experiencing pain. This can be explained partly by the prevalence of musical anhedonia and a general dislike for music among a subset of the global population, but also potentially by age, gender and cultural context. Specifically, a lower willingness to listen to music in healthcare was associated with higher age and female gender in the overall data. With respect to age, our findings align with studies showing that the importance attributed to music tends to decline with increasing age while music tastes consolidate (Bonneville-Roussy et al., 2013), and that younger people listen to more music, more actively, and across spatial contexts (Nowak & Bennett, 2020). Specifically, older individuals show varying attitudes toward music, influenced by generational factors and cultural norms. These norms could be more prominent in healthcare, where older patients more frequently exhibit politeness and respect, and therefore may refrain from requesting or listening to music unless specifically advised by their doctor (Wyman et al., 2018). Additionally, older individuals tend to listen to music in private contexts, which might also explain their lower willingness to listen to music in healthcare (Bonneville-Roussy et al., 2013). With respect to gender, the current literature does not adequately explain our findings. Importantly, people's engagement with music is influenced by their social identities, with gender and age being just a few of many factors.

The preference to (not) listen to music varied among the twenty countries. This variation between national populations is fruitful to discuss, although in this paper, we cannot empirically identify any mechanisms that explain these relationships, as our data do not allow exploration from a cultural perspective. Healthcare practices, individual preferences and differences among subregions at national level may all contribute to these variations, highlighting areas for further research. The percentage of individuals who did not want to listen to music was particularly high in Saudi Arabia, Japan and the UAE. In contrast, in the African countries South Africa and Nigeria, the percentages of individuals who wanted to listen to music were remarkably high. Tentative explanations could be guided by widespread cultural and religious differences. For instance, religious beliefs may shape the attitudes toward music listening (Otterbeck & Ackfeldt, 2012). In addition, variations in the perceptions of pain and pain management strategies across different countries and different ethno-racial backgrounds could also account for some of the differences that were observed (Ashton-James et al., 2023; Covert et al., 2022; Mathur et al., 2020). The necessity for pain management also hinges on whether pain is perceived as a significant problem, which in turn is influenced by cultural and social factors (Fillingim, 2017; Imanpour & Park, 2023; Krahé et al., 2013). Previous studies have revealed various international variations in beliefs about pain and pain-related coping strategies, such as praying, resting, catastrophizing, and seeking social support (Ferreira-Valente et al., 2011). In particular, religious beliefs can influence perceptions and beliefs about pain (Najem et al., 2021; Thong et al., 2017). For example, in Islamic theology, patiently bearing pain has a positive influence on the soul's prospects in the afterlife (Branden & Broeckaert, 2010). Context and pain beliefs are crucial aspects of the potential working mechanisms of music for pain relief (Cohen et al., 2023; Lunde et al., 2019). Hence, cultural context, along with closely linked pain beliefs, can be explanatory factors in interpreting the selection of music versus no music across different countries.

### 4.2 Music genre selection

Prior research highlights the importance of overall music (genre) preference and familiarity with music when looking at music for pain relief (Basiński et al., 2021; Howlin & Rooney, 2021; Valevicius et al., 2023; Van der Valk Bouman et al., 2024). This aligns with our findings, as participants predominantly chose their favorite music genre(s) when experiencing pain (73.1%). This is advantageous, as most people already select the genres that are probably best for them, with previous studies indicating that favorite music works best for pain relief.

Strong alignment between certain music genres and social groups also seems to play a role when we zoom into the USA and UK subsamples of the data. First, regarding socio-economic status, in the USA subsample, participants with higher incomes were more likely to select non-favorite music (which particularly consisted of classical music). This finding might suggest that people from higher status groups could be inclined to align their choices with the cultural belief that classical music is superior, although this observation was not observed in the UK subsample. This interpretation aligns with previous research, which has suggested that people from higher status backgrounds tend to prefer classical music more than people from lower status backgrounds do (Bourdieu, 1984; Lizardo & Skiles, 2016; Van Eijck, 2001). Second, with respect to gender, female participants were more likely to choose non-favorite music than men were. Whereas previous research has indicated that men and women tend to display different taste preferences (Colley, 2008; Savage, 2006), we do not have sufficient data to explain the observed relationship, which deserves further research. Third, we can also consider ethno-racial differences for the US subsample. Previous research has established that individuals of African-American descent overwhelmingly prefer predominantly ‘Black’ music genres such as hip-hop, soul and jazz as a source of empowerment and representation (Clay, 2003; Lizardo & Skiles, 2016; Schaap et al., 2022). Our findings suggest that this may also flow into preferences for music for pain relief, as individuals of African-American descent in the USA had a greater probability of selecting their favorite music genres when experiencing pain (Lizardo & Skiles, 2016).

While the therapeutic value of music for pain relief has been investigated predominantly in the Global North, it is important to note that the aforementioned national variations may also influence the selection of specific music (genres) (Good et al., 2000). This study revealed distinct patterns in music genre selection when experiencing pain between national populations. For example, while pop music was most popular in the Global North, blue note music was the most popular choice in Nigeria and South Africa, and classical music was most popular in Saudia Arabia and the UAE. Further research is needed to explore how this affects music in various international healthcare settings. Noteworthy, in terms of non-favorite music genre selection, classical and mellow music emerged as the most popular choices in nearly all countries. Both classical and mellow music have been frequently used in previous clinical studies (Hole et al., 2015). The high percentage of classical music within the non-favorite genres is in line with the cultural belief that classical music is better at reducing pain than any other music genre is (Garza-Villarreal et al., 2014). Interestingly, this ‘Mozart effect’ seems to transcend borders, as participants in the Global South and North tended to select classical music, even if this was not their favorite music.

Numerous studies on music in healthcare have focused exclusively on classical music, consistently demonstrating its effectiveness in alleviating pain (Choi et al., 2023). However, this superior effect of classical music compared with other music genres has not been proven in the literature. In contrast, a recent study comparing various music genres for pain relief revealed that classical music was not superior to other music genres, but that listening to a favorite music genre had a significant positive influence on pain tolerance, irrespective of the music genre itself (Van der Valk Bouman et al., 2024). Moreover, older participants who chose non-favorite music genres were more inclined to select classical music when experiencing pain. This suggests that the belief in the superiority of classical music may be decreasing among younger generations, potentially shifting toward more mellow genres such as ambient, lo-fi and easy listening. Additionally, the perceptions of older participants might be more strongly influenced by media attention given to the ‘Mozart effect’ in the 1990s. Other factors contributing to these observations could include participants giving socially desirable answers, or feeling that their music taste might be judged by healthcare professionals (Bourdieu, 1984; Lizardo & Skiles, 2016). The preference for classical music among participants may also be influenced by ‘cultural goodwill’; the (unconscious) desire to appear or belong to a higher social class, as individuals from higher social classes generally receive better healthcare compared to those from lower classes. Although we observed the ‘Mozart effect’ across twenty countries, it is important to highlight that only a small percentage of participants (26.9%) chose non-favorite music genres, such as classical music, whereas the majority (73.1%) preferred their favorite music.

### 4.3 Clinical implications

There are several recommendations for clinical practice based on the results of this study. First, healthcare professionals should acknowledge and recognize the cultural belief known as the ‘Mozart effect’. When discussing music with patients, it is essential to provide information that favorite and/or familiar music can be most effective – and that there is no proof that classical music is superior for pain relief. Second, healthcare professionals should approach conversations about music in a culturally sensitive manner, considering both cultural and personal differences. Third, respecting patients' preferences, including the choice of ‘no music at all’, is crucial. It is important to incorporate cultural and individual aspects into (inter)national guidelines about music in healthcare, including the option to not listen to music. Finally, healthcare professionals around the world should prioritize offering patients the music of their choice and providing a wide variety of music genres, similar to those available on music streaming platforms, to cater to diverse musical tastes.

### 4.4 Strengths, limitations and directions for future research

A major strength of this study was the large sample size, with almost 34,000 participants. Moreover, the variety of respondents from twenty culturally diverse countries enhances validity and reliability of the results, with the UK and USA being economically and ethnically representative samples. The first and greatest limitation of this study lies in the basis of the survey method, as the findings are self-reported perceptions. Therefore, we do not know exactly how participants would truly respond when experiencing pain in healthcare. However, these (broader) perceptions are still important, as there remains a lack of knowledge on the general population's perspective in this field. While studies exist that examine true responses to music selection in the context of pain, they often lack patient perceptions and the extensive scale of our study. Thus, our findings complement existing research by providing a broader understanding of the general population's views on music for pain relief. Nevertheless, future clinical trials with experimental designs such as randomized controlled trials should investigate across (culturally diverse) populations to capture true responses to pain, particularly considering individual music preferences and music choices. A second limitation of this study was that the sample of IFPI respondents was relatively young and the 65+ age subgroup was not present for all countries, which may limit the generalizability of our findings to clinical settings with predominantly elderly patients. Because IFPI's panels were representative of the online population, there was a selection bias that likely resulted in fewer elderly individuals being reached. The selection of music for pain relief in especially older patients should be investigated in future research. Third, this study focused on national populations, limiting conclusions on cultural aspects. Specifically, no countries in Central and/or South America were included, restricting the global inclusivity of this study. Furthermore, while race-ethnicity and income data were available for the UK and USA, these findings cannot be generalized to other populations. Besides, the different subgroup categorizations of income and race/ethnicity for the UK and the USA further limit the comparability of the data between these two countries. Future research should explore these questions further. Finally, it is important to note that the survey question was asked as a broad question about general pain, and that we did not know what type of pain or context the participants were thinking of when they answered the question. On one hand, individual pain perceptions vary in terms of nature, intensity and duration. On the other hand, medical scenarios and music listening methods can differ significantly, such as ambient music in waiting rooms or self-selected music via headphones during surgical procedures. Additional research is needed to better understand the context that shapes people's attitudes toward music for pain relief, and to investigate more specific scenarios of different types of pain in different healthcare settings. For instance, studies should explore a wider range of music genres and characteristics (e.g., rhythm, harmony, tempo, and pitch) for pain management, and investigate personalized approaches using artificial intelligence. Moreover, comparing listening preferences for audio other than music, such as prayer, podcasts, and audiobooks, could yield valuable insights in this research field.

## 5 Conclusion

In conclusion, a large majority (86.5%) of the global population is willing to listen to music when experiencing pain in healthcare. Participants predominantly chose their favorite music, with classical music being the most common choice when selecting a genre not aligned with their overall music preference. This finding supports the existence of the ‘Mozart effect’, the cultural belief that classical music is particularly effective when experiencing pain, at least among a smaller, but substantial, part of the population. There were notable differences between national populations and across social groups (gender, age, race-ethnicity, income) in the preference for music when experiencing pain. This knowledge can be crucial in healthcare worldwide, emphasizing the need for a culturally sensitive and personalized approach to utilizing music as medicine.

## CRediT authorship contribution statement

**Antonia S. Becker:** Writing – original draft, Visualization, Methodology, Formal analysis, Conceptualization. **Emy S. van der Valk Bouman:** Writing – original draft, Visualization, Methodology, Formal analysis, Conceptualization. **Julian Schaap:** Writing – review & editing, Supervision, Conceptualization. **Markus Klimek:** Writing – review & editing, Supervision, Conceptualization. **Joost Oude Groeniger:** Writing – review & editing, Supervision, Conceptualization.

## Ethical statement

The data collection and ethical approval for this study were organized by the International Federation of the Phonographic Industry (IFPI). Prior to the survey, informed consent was obtained from all participants. Data were anonymized by the IFPI before being transferred to the researchers to ensure participant confidentiality and privacy. There was no further need to get ethical approval as this study used an anonymized dataset for secondary analysis.

## Declaration of generative AI and AI-assisted technologies in the writing process

During the preparation of this work the authors used Microsoft CoPilot and Curie in order to edit and improve the language. After using these tools, the authors reviewed and edited the content as needed and take full responsibility for the content of the publication.

## Funding sources

This research was financed by the Erasmus MC Foundation. Research time of J.S. was funded by Netherlands Organization for Scientific Research (10.13039/501100003246NWO project #Vl.Veni.211S.116). Additional support was provided by the Erasmus Initiative ‘Smarter Choices for Better Health’ and 10.13039/501100022216Medical Delta. The funders had no role in the study design or the analysis and interpretation of the data. All authors and their institutions reserve intellectual freedom from the funders.

## Declaration of competing interest

The authors declare that they have no known competing financial interests or personal relationships that could have appeared to influence the work reported in this paper.

## Data Availability

The authors do not have permission to share data.
