# Supplementary material for: Music in healthcare: Investigating music preferences for pain management across twenty countries
Source: SSM Popul Health. 2025 Jan 22;29:101758. doi: 10.1016/j.ssmph.2025.101758 (PMC11804819; doi:10.1016/j.ssmph.2025.101758)
Supplement: Multimedia component 1 [file mmc1.docx]

**SUPPLEMENTARY MATERIAL**

Suppl. Table 1 Overview of panel sizes, population sizes, panel age ranges and panel providers per country

| **Country** | **N of survey** | **Total population of country (2023)*** | **Age range within panel (years)** | **Panel provider used for survey data collection** |
| --- | --- | --- | --- | --- |
| Australia | 1535 | 26,439,112 | 16-64 | Prodege |
| Canada | 1542 | 38,781,292 | 16-64 | Prodege |
| China | 3020 | 1,425,671,352 | 16-44 | GMO |
| France | 2042 | 64,756,584 | 16+ | Bilendi |
| Germany | 1502 | 83,294,633 | 16-64 | Bilendi |
| India | 3004 | 1,428,627,663 | 16-44 | Rakuten |
| Italy | 1514 | 58,870,763 | 16-64 | Bilendi |
| Japan | 1593 | 123,294,513 | 16-64 | Rakuten |
| The Netherlands | 1419 | 17,618,299 | 16+ | Bilendi |
| New Zealand | 1111 | 5,228,100 | 16-64 | Probity Research |
| Nigeria | 743 | 223,804,632 | 16-44 | Borderless Access |
| Poland | 1110 | 41,026,068 | 16-64 | JTN |
| Saudi Arabia | 1128 | 36,947,025 | 16-44 | Borderless Access |
| South Africa | 1130 | 60,414,495 | 16-64 | Probity Research |
| South Korea | 1546 | 51,784,059 | 16-64 | Rakuten |
| Spain | 1533 | 47,519,628 | 16-64 | Bilendi |
| Sweden | 1525 | 10,612,086 | 16-64 | Bilendi |
| UAE | 1108 | 9,516,871 | 16-44 | Borderless Access |
| UK | 1524 | 67,736,802 | 16-64 | Bilendi |
| USA | 4000 | 339,996,564 | 16-64 | Prodege |
| Total | 33629 | 4,161,940,541 | see above | see above |

Abbreviations: UAE = United Arab Emirates, UK = United Kingdom, USA = United States of America

**Suppl. Table 2 Overview of the question ‘music as medicine’**

| **Question** | If you were in a hospital or other healthcare setting and experiencing pain, what type of music would you most like to listen to? Please select one choice only. |
| --- | --- |
| **Answer options** | Genre selected as first choice in question about favorite music genre – if different to genre groups below |
|  | Genre selected as second choice in question about favorite music genre – if different to genre groups below |
|  | Genre selected as third choice in question about favorite music genre – if different to genre groups below |
|  | Pop |
|  | Rock |
|  | Classical |
|  | Ambient |
|  | Hip-hop / Rap |
|  | Dance music |
|  | Other (please specify)  Free text option |

**Suppl. Table 3 Answer options of demographic characteristics**

| **Category** | **Answer options** |
| --- | --- |
| Age | Number in years |
| Gender  (single choice) | Female  Male  Not listed/other  I prefer not to answer |
| Ethnicity UK  (single choice) | White British  White European  White - Other  Indian  Pakistani  Bangladeshi  Chinese  Japanese  Any other Asian/Asian British Origin  African  Caribbean  Any other Black/African/Caribbean British origin  Arab  Any other ethnic origin  White and Black Caribbean  White and Black African  White and Asian  Any other Mixed/Multiple ethnic origins  Prefer not to say |
| Ethnicity US  (multiple choice) | White  Hispanic, Latino or Spanish  Black or African American  Asian  American Indian or Alaskan Native  Native Hawaiian or other Pacific Islander  Another race or ethnicity  Prefer not to say |
| Income UK  (single choice) | Less than £10,000  £10,000 - £19,999  £20,000 - £29,999  £30,000 - £39,999  £40,000 - £49,999  £50,000 - £59,999  £60,000 - £69,999  £70,000 - £79,999  £80,000 - £89,999  £90,000 - £99,999  £100,000 - £149,999  £150,000 - £199,999  £200,000 - £249,999  £250,000 or more  Don't know  Prefer not to say |
| Income US  (single choice) | Less than $10,000  $10,000 to $14,999  $15,000 to $24,999  $25,000 to $34,999  $35,000 to $49,999  $50,000 to $74,999  $75,000 to $99,999  $100,000 to $149,999  $150,000 to $199,999  $200,000 or more  Don’t know  Prefer not to say |

Abbreviations: UK = United Kingdom, USA = United States of America

**Suppl. Table 4** **Overview of genre groups with subgenre categorization**

| **Genre group** | **Subgenres within genre groups** |
| --- | --- |
| Blue Note | Blues; Gospel; Jazz |
| Classical | Classical; Opera |
| Country | Americana; Bluegrass; Country |
| (Electronic) Dance | Electronic; EDM; Dance; Drum and Bass; Hardstyle; House; Techno; Trance |
| Folk | Folk |
| Global | Afrobeats; Bollywood; Latin; Regional music (e.g. African/Arabic/Dutch/ French/Indian/Swedish music) |
| Mellow | Ambient; Easy Listening; Lo-fi; Instrumental |
| Pop | Decades (e.g. 60 s/70 s/80 s music); (Local) Pop; Singer-songwriter |
| R&B | Funk; R&B; Soul |
| Rap/hip-hop | Hip-hop; Rap |
| Reggae | Reggae; Ska |
| Religious | Religious music (e.g. Buddhist/Christian/Islamic music) |
| Rock | Alternative/indie; Gothic; Heavy Metal; Metal; Punk; Rock |
| Soundtracks | Musicals; Movie or TV; Soundtracks |
| Utility | Binaural Beats; Calming; Healing Frequencies; Meditation; Nature Sounds; New Age; Relaxation; Spa; Stress Relief; Yoga; Zen |
| Vocal | Celebratory; Children; Choral; Christmas; Holiday; Sing-Along; Schlager |
| Other | Audiobooks; Mix; Radio; Specific artists; Undefinable; White Noise |

Abbreviations: EDM = Electronic Dance Music, TV = Television

Suppl. Table 5 Baseline characteristics of the United Kingdom and the United States of America

|  | | **UK  (n=1524)** | **USA  (n=4000)** |
| --- | --- | --- | --- |
| **Age -** Mean (SD) | | 39.99 (13.99) | 40.48 (13.78) |
| **Female** - % (n) | | 50.82 (772) | 50.75 (2027) |
| **Race-ethnicity**^a^ - % (n) | |  |  |
| White | | 84.19 (1267) | 65.58 (2610) |
| Black | | 3.79 (57) | 13.82 (550) |
| Asian | | 9.50 (143) | 6.13 (244) |
| Hispanic/Latino | |  | 5.05 (201) |
| Mixed | | 1.73 (26) | 8.24 (328) |
| Other | | 0.80 (12) | 1.18 (47) |
| **Income^b^** (UK /USA) - % (n) | |  |  |
| Less than £20,000 | / $24,999 | 19.37 (265) | 19.24 (754) |
| £20,000 - £39,999 | / $25,000-49,999 | 37.43 (512) | 24.68 (967) |
| £40,000 - £59,999 | / $50,000-74,999 | 22.22 (304) | 20.55 (805) |
|  | / $75,000-99,999 |  | 13.17 (516) |
| More than £60,000 | / $100,000 | 20.98 (287) | 22.36 (876) |

^a^ UK: N=19 missing; USA: N=20 missing (Prefer not to say); ^b^ UK: N=156 missing; USA: N=82 missing (Prefer not to say/Don’t know).

Abbreviations: UK = United Kingdom, USA = United States of America

**Suppl. Figure 1 Top five music choices when experiencing pain by country (%)**


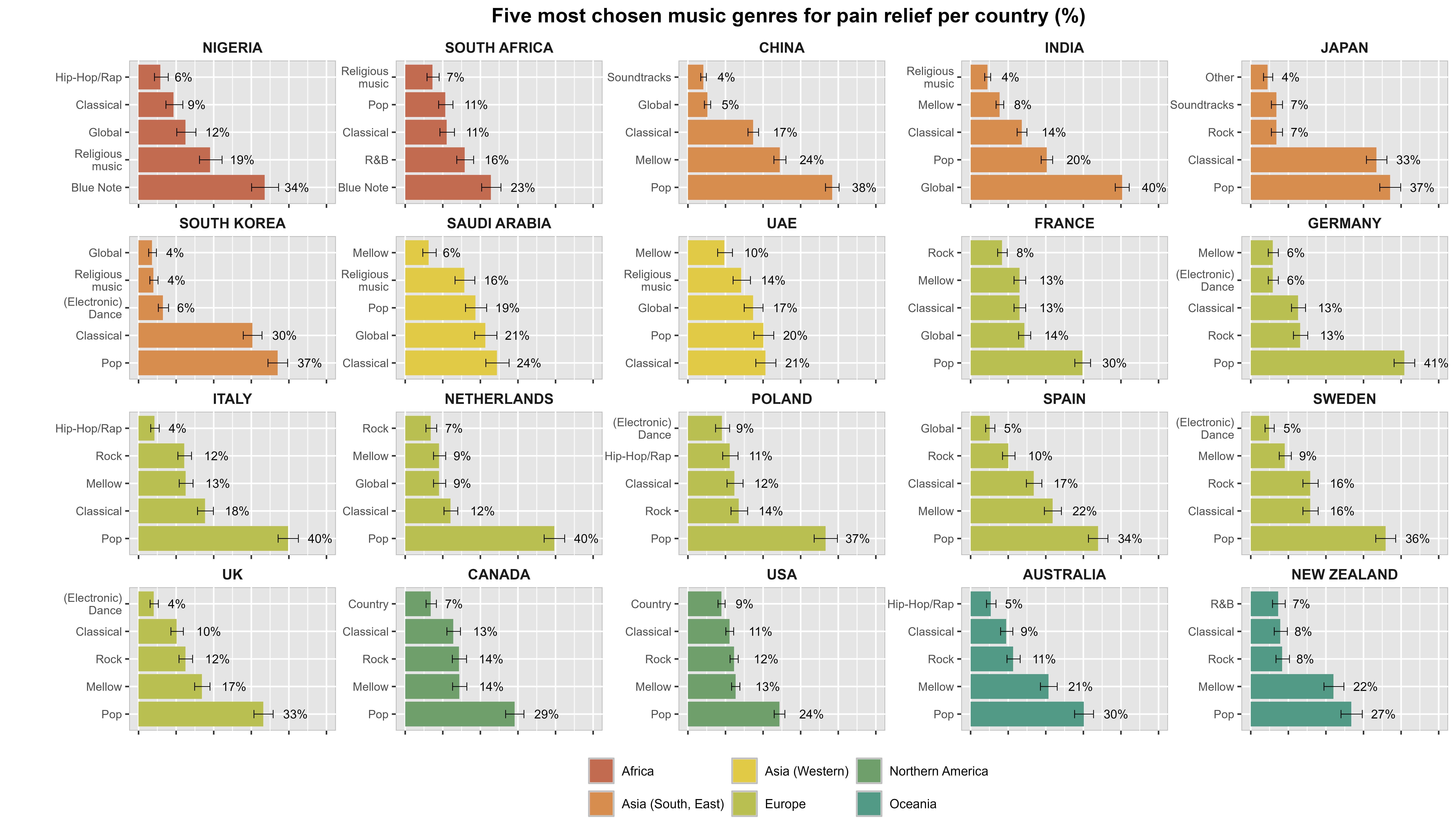


Abbreviations: UAE = United Arab Emirates, UK = United Kingdom, USA = United States of America

**Suppl. Figure 2 Top five music genres under ‘Non-favorite’ by country (%)**


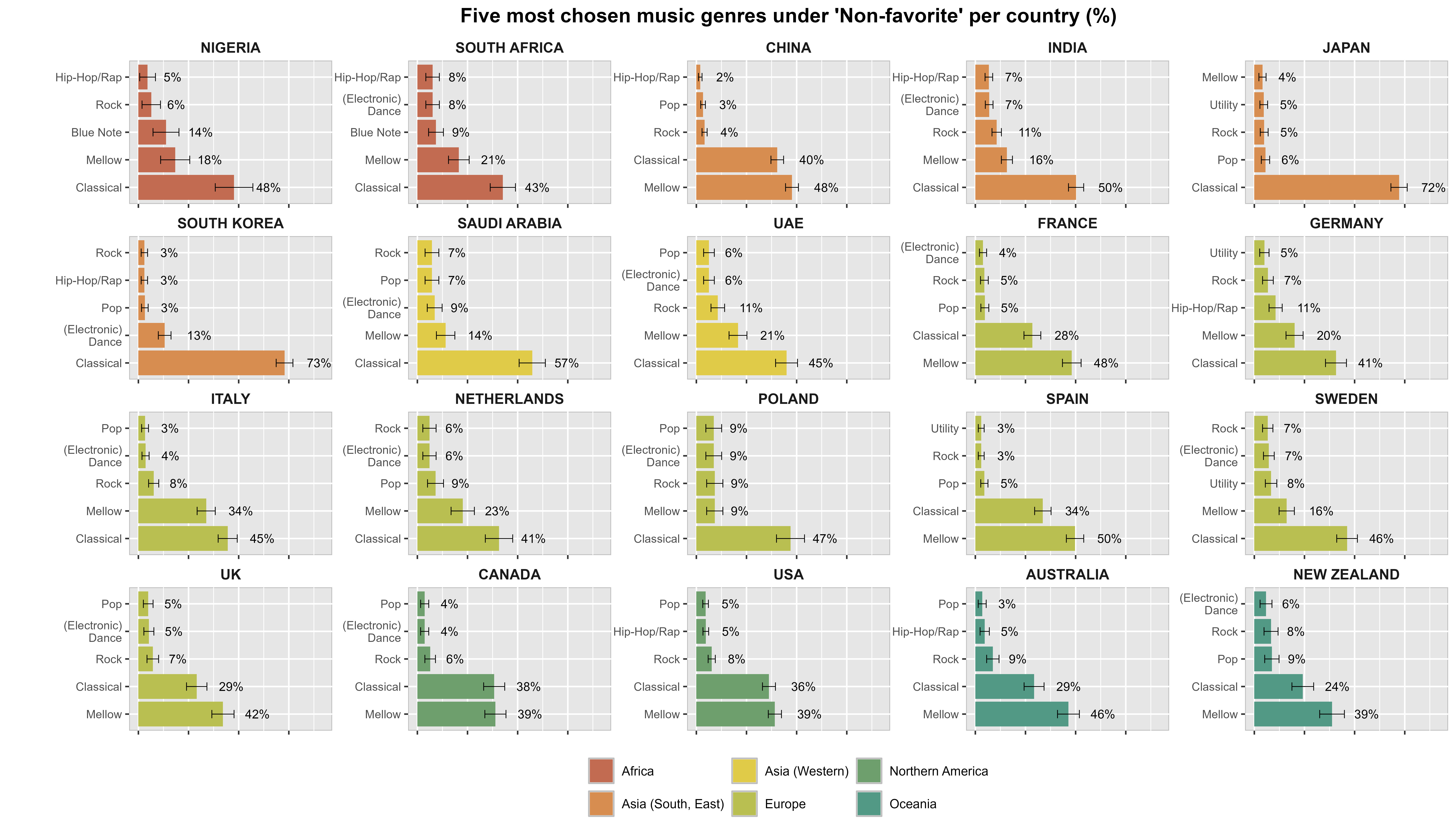


Abbreviations: UAE = United Arab Emirates, UK = United Kingdom, USA = United States of America
